# A RE-AIM evaluation in early adopters to iteratively improve the online BeUpstanding™ program supporting workers to sit less and move more

**DOI:** 10.1186/s12889-021-11993-1

**Published:** 2021-10-22

**Authors:** Genevieve N. Healy, Elisabeth A. H. Winkler, Ana D. Goode

**Affiliations:** 1 The University of Queensland, School of Public Health, 288 Herston Rd, HERSTON QLD, Brisbane, 4006 Australia; 2 Baker Heart & Diabetes Institute, Melbourne, Australia

## Abstract

**Background:**

The web-based BeUpstanding program supports desk workers to sit less and move more. Successfully translated from a research-delivered intervention, BeUpstanding has gone through iterative development and evaluation phases in preparation for wide-scale implementation. In the third planned “early-adopters” phase (01/09/2017–11/06/2019), the program was made freely-available online. An integrated delivery and evaluation platform was also developed to enable workplace champions to run and evaluate the intervention within their work team independent of researcher support. Using the RE-AIM (Reach, Effectiveness, Adoption, Implementation, Maintenance) framework, this study reports on the extent to which the program and processes were “fit-for-purpose” for a national implementation trial across the indicators of uptake (reach and adoption), implementation and engagement, and effectiveness for behaviour change.

**Methods:**

Data were collected via the online surveys embedded in the program and through program access analytics. Descriptive data (with linearized variance for the clustered staff-level data) and results from mixed models (repeated data and clustering for pre-post changes) are reported.

**Results:**

Despite purposeful limited promotion, uptake was good, with 182 Australian users initially registering (208 total) and 135 (from 113 organisations) then completing the sign-up process. Recruitment reached users across Australia and in 16 of 19 Australian industries. Implementation was inconsistent and limited, with signed-up users completing 0 to 14 of the program’s 14 steps and only 7 (5.2%) completing all seven core steps. Many champions (*n* = 69, 51.1%) had low engagement (1 day toolkit usage) and few (*n* = 30, 22%) were highly engaged (> 1 day toolkit usage and surveyed staff). Although only 18 users (7 organisations) performed the pre- and post-program staff evaluations (337 and 167 staff, respectively), pre-post changes showed the program effectively reduced workplace sitting by − 9.0% (95% CI -12.0, − 5.9%).

**Discussion:**

The program had uptake across industries and across Australia, but implementation and engagement varied widely. Few workplaces completed the evaluation components. In those that did, the program was effective for the primary outcome (workplace sitting). Conducting a planned early adopters phase and a comprehensive evaluation according to RE-AIM helped highlight necessary program improvements to make it more suitable for wide-scale implementation and evaluation.

**Trial registration:**

Australian and New Zealand Clinic Trials Registry ACTRN12617000682347. Date registered: 12/05/2017.

**Supplementary Information:**

The online version contains supplementary material available at 10.1186/s12889-021-11993-1.

## Introduction

High levels of sitting are associated with morbidity and premature mortality, with adults who are also physically inactive at the greatest risk [1, 2]. The desk-based workplace has been identified as a key setting in which to address this ubiquitous health behaviour, with desk workers typically spending 70–80% of their workday sitting [3]. Several studies have now demonstrated that reducing sitting time in this setting is feasible and acceptable to both employers and employees [4, 5]. Efforts to address this behaviour are being supported by regulators and key industry and advocacy bodies [6–9], with prolonged workplace sitting identified as an emergent work health and safety issue [6].

The BeUpstanding™ program and accompanying BeUpstanding Champion Toolkit [10] were developed to support the wide-scale uptake and implementation of evidence-based strategies to reduce workplace sitting. The BeUpstanding program is based on the flagship *Stand Up Australia* program of research [11] and was developed in collaboration with key national and state policy and practice partners [12]. Details of the web-based program and toolkit are provided elsewhere [13], but in brief, the toolkit provides a step-by-step guide with associated multi-media resources (e.g., videos, email templates, posters, survey links) to assist a workplace champion to implement the BeUpstanding program within their own work team. Over a period of two to three months, the program focusses on raising staff awareness of the benefits of sitting less and building a supportive team culture to create behaviour change.

Designing for dissemination often requires an iterative process of development and adaptation to ensure the resulting product is suitable for implementation in ‘real-world’ contexts [14, 15]. However, systematic documentation and reporting of how this dissemination process unfolds, and the iterative phases of development and testing, is rarely reported [16, 17]. To contribute to this evidence base, the development of the BeUpstanding program and the associated online delivery platform has gone through multiple planned phases [12], with findings and learnings from each phase reported. This iterative process has also been grounded in the RE-AIM (Reach, Effectiveness, Adoption, Implementation, Maintenance) framework [18] due to its intuitiveness in helping both design and evaluate programs in applied contexts [19]. A summary of the Phases is shown in Table 1.

**Table 1:** The iterative development and evaluation phases of the BeUpstanding™ program with Phases 2–5 guided by the RE-AIM framework

Phase (Timeline)	Primary aims of phase
**Phase 1** (c. 2015–2016) “Adaption and adoption”	• To adapt the *Stand Up Australia* intervention [11] to a low cost/no cost program with the ability to scale-up • To develop research-government partnerships
**Phase 2** (c. 2016–2017) “Beta-testing”	• To pilot test the Beta-version of the BeUpstanding program and toolkit in a targeted sample of workplaces for feasibility and acceptability • To determine the feasibility and acceptability of the “train-the-champion” approach
**Phase 3** (c. 2017–2019) “Early-adopters”	• To test the on-boarding (sign-up) process and the new implementation and evaluation platform • To evaluate the extent to which the program and processes were “fit-for-purpose” for the planned national implementation trial
**Phase 4** (c. 2019–2022) “National implementation trial”	• To evaluate the uptake, implementation, effectiveness, and maintenance of the program in the context of a national implementation trial • To determine the costs and outcomes of scaling up to national implementation, including intervention affordability and sustainability.
**Phase 5** (c. 2021-ongoing) “Optimisation and ongoing evaluation”	• To integrate the learnings from the national implementation trial • To evaluate the optimised program, including sustainability

Phase 1 involved the initial development of the ‘BeUpstanding Champion Toolkit’ and formation of research-government partnerships [12]. In this Phase, the focus was on adapting the original intervention material and protocols from *Stand Up Australia* [20] to something more suitable for wide-scale delivery. The primary adaptation was the transfer of responsibility for program delivery and evaluation from the research team to a workplace champion. Correspondingly, this neccesitated the development of associated training content and program materials delivered via an online toolkit to help guide the champion. Program content was tailored to be more consumer friendly for both champions delivering and evaluating the program, and staff exposed to program messages.

In Phase 2, a beta (test) version of the BeUpstanding program was evaluated, with the findings demonstrating that the “train-the-champion” approach used within the program was highly acceptable, feasible to implement, and effective in reducing self-reporting workplace sitting time [21, 22]. The test version of the program in Phase 2 focused on the feasibility of non-researcher led implementation: it was not designed to be easily adopted and evaluated by workplaces without researcher support. Champions were recruited by the researcher team, with the program resources and materials accessed via a simple, password-protected website, and an external tool used for the survey evaluations. To be able to deliver and evaluate the program at scale, there needed to be mechanisms through which champions find out and adopt the program, as well as evaluate and share their progress and findings with the research team without their direct involvement. For BeUpstanding, these mechanisms developed for Phase 3 included informational boarding pages and associated collateral, as well as the integration of evaluation components for the first time within the toolkit via a bespoke technology platform, with the intention that pre- and post-program evaluation became a ‘core component’ for champions to lead as part of the delivery of the program to staff.

The updated website and toolkit went live in a “soft-launch” on September 1st, 2017 and was provided freely accessible to workplaces across Australia, but without support from the research team and with only limited promotion through the established research-government partnership avenues. The purpose of this phase (Phase 3) was to test the new onboarding and recruitment channels and the new integrated delivery and evaluation platform. The ultimate goal of this phase was to inform further optimisation of the program prior to its’ evaluation in the context of a national implementation trial (Phase 4 [13]). Participants who took part during Phase 3 were known as “early adopters”.

This current study focuses on Australian early adopters, reporting the extent to which the program was working as intended according to the RE-AIM framework [18]. Specifically, adoption and reach are described in terms of the characteristics of those using the toolkit, their organisations and the workers to whom BeUpstanding is to be delivered (i.e., their teams); implementation is reported in terms of the program steps performed and the engagement of users with the online toolkit; and, program effectivess is reported for the primary outcome (workplace sitting) as well as its potential replacement with standing and moving. Maintenance was not measured in this phase. This study also reports on the implications of the early adopters findings for improving the next iteration of the toolkit to make it suitable for the planned national implementation trial (Phase 4), focusing on both the program delivery and data collection.

## Methods

### Study design and recruitment

The study uses a hybrid implementation-effectiveness design, focusing here on a quantitative description of the reach, adoption, implementation and engagement of the BeUpstanding toolkit (early adpopters version) with effectiveness evaluated via a single-group pretest-posttest design [23]. There was deliberate low-scale promotion of the program during the early adopters phase, with partners asked to limit their promotional activities to one promotional channel initially (e.g., a workshop; a member e-newsletter). All valid Australian-based users who registered for the BeUpstanding program from the 1st September 2017 (the day the toolkit went “live”) to June 11th, 2019 (the day prior to the major upgrade based on the learnings from this early adopters phase) were included. Data were extracted from the toolkit on the 7th May 2021, with this lag-time allowing for an appropriate duration of data capture. All users were considered “valid” unless they were ascertained to be using the toolkit for reasons other than running BeUpstanding (i.e., members of the research team and their contacts) or students / journalists / bloggers with fictitious businesses. The study was approved by the University of Queensland’s Human Research Ethics Committee (#2016001743) and conducted in accordance with the Declaration of Helsinki, with toolkit users and staff providing informed online consent prior to participating in any data collection. The study was prospectively registered with the Australian and New Zealand Clinic Trials Registry (ACTRN12617000682347. Date registered: 12/05/2017).

### The BeUpstanding program: early adopters version

**Fig. 1:**
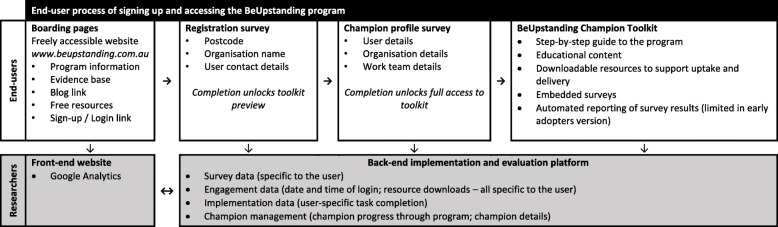
The process for end-users and researchers of the BeUpstanding program

The aim of the BeUpstanding program is to create a more dynamic workplace through raising awareness of the benefits of sitting less and moving more and creating a supportive culture for change [12, 13]. In brief, BeUpstanding uses a “train-the-champion” approach, where a champion (designated by the workplace) uses the toolkit and associated resources and materials to deliver the BeUpstanding program to their work team. In the early adopters version, the user first registered for the program then unlocked the online toolkit by completing a survey, upon which they had access to all of the toolkit resources. These included the multimedia downloadable resources and the step-by-step instructions that were intended to guide the champion through delivering the program in three phases: Plan (gain support from management, perform a needs assessment, formulate an action plan); Do (promote the key messages through emails and posters for > 4 weeks, ideally 8–12 weeks); and, Review (evaluation surveys, reflect, celebrate success). The toolkit provided the resources required to perform these steps, including embedded surveys to help the champion understand the current policies, practices and behaviors of their work team. Of the 14 steps involved in the toolkit, half (*n* = 7) were considered core (i.e., they were deemed essential by the research team for appropriate implementation of the program) with the other half considered optional depending on the needs of the team and organisational requirements. The core elements spanned the ‘Plan, Do, Review’ phases and included: the staff pre- and post-program surveys; the pre- and post-program workplace audit; staff education and consultation on team strategies to sit less and move more; and, promotion of program messages and team strategies through emails and posters. The end-user journey through the sign-up process through to the toolkit, and how this integrates with the front-end boarding pages and the back-end technology platform accessed by the researchers, is shown in Fig. 1.

### Data collection, measures and outcomes: early adopters version

Data were collected through the BeUpstanding toolkit through the implementation platform underpinning the BeUpstanding program [13]. Data were collected from users via web entry (registration information and completion of program steps), via online user-completion surveys (profile survey, pre- and post-program workplace audits), user-disseminated surveys for staff to complete (pre- and post-program staff surveys), and via passive data collection of website usage through the website (file downloads). Champion surveys were identified to each user through their login. Staff surveys were anonymous, but identifiable to the champion who had distributed the staff surveys. To match pre- with post- staff responses while preserving anonymity, an identifier comprised of organisation, initial of mother’s first name (A–Z), birth day of month (0–31), and last three digits of mobile phone (000–999) was constructed, with year of birth and sex further used to discriminate between different individuals if any non-unique identifiers emerged. All repeated responses from the same staff member were discarded. This study reports on selected RE-AIM [18] indicators that reflect the extent to which the program was working as intended. The data collection methods of the early adopters version of the toolkit are critiqued, with the relevant improvements required (and made) to provide a more robust evaluation for the planned national implementation trial highlighted.

#### Adoption and reach

Indicators of adoption and reach (uptake) were the number of toolkit users (champions) and the organisations and workers (team members) they collectively represent, as well as their characteristics. Of particular importance was ensuring diverse recruitment geographically and across industries and sectors. Key partner-identified sectors were blue collar, small business, and government/public sectors as well as teams from regional/remote locations and call-centre workplaces. Additionally reported elements were organisation size, and characteristics of champions (age, sex, job category, expertise relevant to championing the program) and their work teams as perceived by the champions (team size, proportion who do predominantly desk based work, proportion of staff in high sitting roles) and as reported by staff (age, sex, education, exercise level [24], job classification, shift work status, hours worked per week, and desired percentage of time spent sitting, standing and moving at the workplace). The referral pathway into BeUpstanding was also reported. Organisation size, industry, and sector status were sometimes reported differently by champions from the same organisation. Conflicting organisational size and government/public status were recoded based on publicly available information while responses on the other traits were treated as different perspectives on the team’s niche within the organisation and permitted to vary.

#### Implementation and engagement

Program implementation was considered in terms of completion of program steps and progress through the pre-program and program phases (Plan, Do, Review). Steps that could not be verified were treated as performed if the champion reported doing the step. Steps that could be verified (i.e., champion-entered surveys) were only considered performed if a completed survey was acquired through the toolkit. Because a champion could have sent out staff surveys without receiving any completed responses, the pre- and post-program staff survey steps were considered completed if the champion reported having done that step, or at least one staff survey was acquired, with a very minimal requirement that the staff member had filled in the anonymous identifier questions at the beginning of the survey. Champions could perform steps in any order (and skip steps). Accordingly, champion progress through the phases was classified by the most advanced step performed. Champion engagement was considered in terms of their usage of the toolkit — all logins and all resource downloads (while logged in) – and evidence staff had been engaged (through the user or another user from their combined team performing pre- and/or post-program staff surveys). Three categories of engagement were created: 1) low: did not access the toolkit beyond the day they unlocked it; 2) moderate: repeat toolkit usage (> 1 day) but did not survey staff; and, 3) high: repeated toolkit usage (> 1 day) and surveyed staff.

#### Effectiveness

The primary effectiveness outcome was self-reported percentage of the workday spent sitting, collected in the pre- and post-program staff surveys using the Occupational Sitting and Physical Activity Questionnaire (OSPAQ [25];). This instrument also captures the percentage of the workday spent standing, walking and in heavy labour and has shown acceptable measurement properties for desk-based workers [25, 26]. Heavy labour is rare in office settings and was thus combined with walking to form ‘moving’. When available items summed to 100%, missing items were replaced with 0% and when available items did not sum to 100%, either the total was recalibrated to sum to 100% (when no item was missing), otherwise the record was excluded.

### Quality of data collection

The quality of the data collection in Phase 3 was evaluated according to the RE-AIM framework [18] to determine the extent to which it was “fit-for-purpose” for the planned Phase 4 national implementation trial [13]. Data collection quality for each indicator was classified subjectively by two raters (GNH and ADG) into three categories: (1) unable / very difficult to collect; (2) collected but incomplete or difficult to collect; or, (3) successfully collected / fit for purpose for the implementation trial. These indicator ratings were then mapped alongside associated improvements made for Phase 4.

### Analyses

Analyses focus on legitimate toolkit users who signed up during the early adopters phase (*n* = 208) who were from Australian workplaces (*n* = 182) and who completed the profile survey required to unlock the toolkit’s features (*n* = 135 users from *n* = 113 organisations). Data were extracted from the toolkit (07 May 2021). Adoption and reach were reported using descriptive statistics, considering biases in participation and retention via logistic regression or mixed logistic regression models accounting for staff nested in clusters (combined teams). Implementation was described per user and per combined team (treating everything done as soon as the first user from the group had done it). Engagement was described overall and plotted over time according to the program phase the champion was currently in. For effectiveness, changes in outcomes between pre- and post-program assessments were analysed using mixed models with random intercepts for cluster (combined team) and participant (repeated measures), and a fixed effects of time (pre / post). Staff members could only reliably be linked to an organisation’s combined team, not the specific champion, because users sometimes surveyed the staff members of a different champion. Only organisations that obtained both pre- and post-program staff surveys were included in the effectiveness analyses, using responses from staff members who provided data at the pre- and/or post-program surveys. A sensitivity analysis adjusted for staff characteristics that were imbalanced between pre- and post-program survey response groups. Analyses were performed in STATA v 16.0 (StataCorp, Texas USA). Significance was set at *p* < 0.05 (two-tailed). Clustering was accounted for using linearized variance estimation (descriptive statistics) or mixed models.

## Results

### Adoption and reach

The uptake of the program was nationwide, with the 182 valid Australian users signing up coming from all Australian states and territories. A further 26 international users signed up, with sign-ups from the United Kingdom (13), various European locations (6), the United States (4), Canada (1), Fiji (1), and Vietnam (1). In total 135 valid Australian users from 113 organisations unlocked the toolkit by completing the champion profile survey. These users collectively claimed a total of 23,154 workers in their teams. Users who registered (n total, n unlocked) were mostly from Queensland (62, 50), Western Australia (40, 26), New South Wales (33, 24), Victoria (21, 13), and South Australia (16, 14) while a small number were from Australian Capital Territory (6, 5), Northern Territory (3, 3), and Tasmania (2, 0). Referral into the BeUpstanding program was primarily through wide-reach promotional avenues (*n* = 31 online, *n* = 14 publications, n = 3 email) and word of mouth (*n* = 41 colleague, *n* = 7 other word of mouth), with some reporting recruitment through presentations (*n* = 15) and assorted other means (*n* = 23), with n = 1 not providing referral information.

Penetration into each industry and sector is shown in Table 2. There was some participation from each of the partner-identified key sectors and from 16 of the 19 industry classifications as defined by the Australian Bureau of Statistics [27]. The greatest inclusion was from Health Care and Social Assistance, Public Administration and Safety, and the Education and Training industries. Many teams (*n* = 90, 66.7%) and organisations (*n* = 68, 60.2%) were from the government / public sector, collectively representing > 10,000 team members. Similarly, champions of regional/remote teams (*n* = 42, 31.1%) reported > 10,000 team members, while the call centre (*n* = 23, 17.0%), blue collar (*n* = 27, 20.0%) and especially small business (n = 23, 17.0%) sector teams reported representing smaller numbers of workers. Organisations (*n* = 113) showed an approximately even mix of champion-reported sizes, with 23 (20.4%) reported as small (< 20 employees), 31 (27.4%) as medium (20–199 employees), 35 (31.0%) as large (200–1999 employees), and 24 (21.2%) as very large (≥2000 employees).

**Table 2:** Penetration into industries and sectors (as identified by user) in the early adopters phase

	Teams reached (n = 135)	Team members reached ^**a**^ (n = 23,154)
**Sector**		
Call centre workplace, yes	23 (17.0%)	3370
Workplace in regional / remote location, yes	42 (31.1%)	14,612
Blue collar, yes	27 (20.0%)	3337
Government / public, yes	90 (66.7%)	17,412
Small business, yes	23 (17.0%)	232
**Industry** ^**b**^		
Accommodation and Food Services	1 (0.7%)	50
Administrative and Support Services	11 (8.1%)	867
Arts and Recreation Services	3 (2.2%)	17
Construction	2 (1.5%)	103
Education and Training	15 (11.1%)	2828
Financial and Insurance Services	5 (3.7%)	116
Health Care and Social Assistance	49 (36.3%)	10,663
Information Media and Telecommunications	2 (1.5%)	102
Manufacturing	2 (1.5%)	208
Mining and Quarries	1 (0.7%)	10
Other Services	6 (4.4%)	454
Professional / Scientific and Technical	13 (9.6%)	1665
Public Administration and Safety	20 (14.8%)	2619
Retail Trade	1 (0.7%)	5
Transport / Postal and Warehousing	3 (2.2%)	3412
Wholesale Trade	1 (0.7%)	35

Table shows n (%)

^a^ Each toolkit user is treated as a champion having one team; team members = sum of team sizes

^b^ As reported by user (recoded when conflicting). Not represented: Agriculture, Forestry and Fishing; Electricity, Gas, Water and Waste Services; and, Rental, Hiring and Real Estate Services [27]

Table 3 shows characteristics of toolkit users, their teams and team members. The obtained group of toolkit users (*n* = 135) were largely but not wholly the user group for which the toolkit was designed, namely workplace champions delivering the intervention to teams of workers who are highly sedentary while doing desk-based work. Users (‘champions’) were mostly female (74.8%) with a mean age of 41.6 years (SD = 11.1). There were 110 (81%) champions who reported at least one form of experience relevant to delivering BeUpstanding: an occupational health and safety role within their organisation (67%); prior training in workplace health promotion (64%); and/or previous experience in delivering a health promotion program (50%). Most self-identified as employees (48.9%) or middle management (41.5%) rather than senior management (9.6%). Six toolkit users (4.4%) reported a team size of zero, suggesting that they were not intending to act as program champions to a specific team, either at all or at the time of filling in the champion profile survey. The remaining 129 champions reported team sizes varying widely from 2 to 8600. Collectively, of the 135 Australian teams, 109 (80.7%) would meet some basic screening criteria to participate in the implementation trial (team size ≥5 and desk-basked work). Nearly all users (88.1%) indicated their teams mostly did primarily desk-based work, and most (78.5%) indicated the majority or more had job roles involving high amounts of sitting. In support of the champion’s perceptions, the staff also reported workplace sitting that on average (M ± SD) was high (73.4 ± 20.2% of their workday), and notably much higher than their levels of desired sitting (43.1 ± 19.7% of their workday).

**Table 3:** Characteristics of early adopters of the BeUpstanding program

Characteristics	n (%) or M ± SD			Difference
	All	Evaluation group ^**a**^		***p*** ^**b**^
**Users (champions)**	**(n = 135)**	**(n = 18)**		
Age, years	41.6 (11.1)	47.1 (12.2)		0.028
Female, yes	101 (74.8%)	14 (77.8%)		0.756
OHS role in workplace, yes	90 (66.7%)	8 (44.4%)		0.037
Experience delivering workplace health promotion program, yes	67 (49.6%)	5 (27.8%)		0.054
Training in workplace health promotion, yes	86 (63.7%)	9 (50.0%)		0.199
*Job classification*				0.754
Employee	66 (48.9%)	10 (55.6%)		
Team leader / Middle management	56 (41.5%)	7 (38.9%)		
Senior management / Executive	13 (9.6%)	1 (5.6%)		
**Team characteristics** ^**c**^	**(n = 135)**	**(n = 18)**		
*N Employees per team*	23,154 Total	702 Total		0.328
0	6 (4.4%)	1 (5.6%)		
1–4	9 (6.7%)	0 (0.0%)		
5–10	28 (20.7%)	6 (33.3%)		
11–19	19 (14.1%)	3 (16.7%)		
20–49	28 (20.7%)	3 (16.7%)		
≥50	45 (33.3%)	5 (27.8%)		
Majority do primarily desk-based work, yes	119 (88.1%)	17 (94.4%)		0.390
*Proportion in high siting job roles*				0.539
The minority	7 (5.2%)	0 (0.0%)		
About half	18 (13.3%)	1 (5.6%)		
The majority	49 (36.3%)	7 (38.9%)		
Nearly all / all	61 (45.2%)	10 (55.6%)		
Meet basic criteria for implementation trial ^d^	109 (80.7%)	17 (94.4%)		0.146
**Team members (staff)**	**(Pre** ***n*** **= 439)**	**(Pre** ***n*** **= 362)**	**(Post** ***n*** **= 175)**	
Age, mean (SD)	44.0 (11.7)	44.4 (11.5)	43.4 (12.9)	0.211
Female, % (n)	362 (82.8%)	305 (84.5%)	146 (83.4%)	0.200
University qualified, % (n)	264 (60.4%)	225 (62.3%)	122 (69.7%)	0.539
Exercise at least 5 days a week, % yes (n)	101 (24.9%)	87 (25.8%)	47 (28.1%)	0.212
*Employment*				0.852
Full time	263 (60.2%)	214 (59.3%)	106 (60.6%)	
Part time	160 (36.6%)	133 (36.8%)	55 (31.4%)	
Casual	14 (3.2%)	14 (3.9%)	14 (8.0%)	
*Reported workplace physical activity*				
Sitting %	73.4 (20.2)	74.0 (19.5)	64.7 (22.3)	0.543
Standing %	14.8 (15.0)	15.2 (15.3)	22.0 (18.4)	0.391
Moving %	11.8 (11.4)	10.8 (9.8)	13.3 (11.7)	0.029
*Desired workplace physical activity*				
Sitting %	43.1 (19.7)	43.5 (19.9)	42.0 (20.6)	0.298
Standing %	31.1 (16.4)	31.5 (16.6)	32.2 (16.2)	0.082
Moving %	25.8 (15.7)	25.0 (15.6)	25.8 (16.5)	0.033

Table reports n (%) or M ± SD OHS = occupational health and safety

^**a**^ Included in the evaluation of effectiveness (organisation received responses to both pre and post staff surveys)

^**b**^ p for difference between those included vs excluded from evaluation group (user-, team- and pre-survey staff characteristics). Tests were from logistic regression or mixed logistic regression models accounting for staff nested in ‘organisational teams’ (i.e., teams from the same organisation combined)

^**c**^ User-reported team characteristics, treating each user as having one separate team

^d^ Screening criteria for implementation trial: Australian, team size ≥5, and majority of team do primarily desk-based work

There was some evidence of bias in participation in the evaluation. The group who went on to complete the evaluation had staff with significantly lower levels of moving and higher levels of desired moving (Table 3). They also had champions who were significantly older and less likely to have an OHS role relative to their non-participating counterparts, but otherwise had similar champion, team, and staff traits. Within the evaluation group, the average age was slightly higher at the pre- than post-program survey (44.4 ± 11.5 vs 43.4 ± 12.9 years, *p* = 0.018), suggesting an underrepresentation of older workers in the post-program survey, but there were no large or significant (*p* < 0.05) difference between pre- and post-program surveys in other sociodemographic attributes or desired behaviours.

### Implementation

Table 4 reports on the implementation of the program and the resources downloaded to support the implementation. The completion rates of each step were highly variable. The most commonly performed step was to conduct a workplace audit (done via an online survey), which was performed by just over half of champions and combined teams. The other early Phase 1 planning steps were performed by a quarter or more champions and combined teams. From the step ‘create and maintain a support network’ onwards, the step completion rates were much lower: 3.7 to 14.6% of champions and 3.5 to 10.6% of combined teams. Some of the champions (35.6%) and combined teams (40.7%) had stopped prior to the planning phase, and most stopped during the program (typically in the planning phase). Very few champions (17.0%) and teams (10.6%) completed the program by reaching the final review phase. Implementation of all the seven core elements was only done by 7 (5.2%) champions and 6 (5.3%) of the combined teams. Each resource was downloaded by anywhere between 7.4 and 64.4% of champions and 7.1 to 67.3% of combined teams, with the most downloaded materials being the program overview, resources for the earliest steps (business case template, sample policy, sample staff emails), tips for staff, and posters. The number of steps completed (Additional File 1) was low, with many champions performing no steps (48/135; 36%) or just one step (27/135; 20%).

**Table 4:** Steps performed and materials downloaded by each individual champion and by at least one champion for each combined team

Phase	Step performed (Early Adopters Version)	Champions n (%)	Teams n (%)
Unlock toolkit	0. Complete a champion profile survey	n = 135	n = 113
Plan Phase (Steps 1–3)	1.1 Make a case for BeUpstanding	44 (32.6%)	41 (36.3%)
	1.2 Formalise commitment in writing	37 (27.4%)	35 (31.0%)
	**2.1 Conduct a workplace audit**	71 (52.6%)	58 (51.3%)
	2.2 Conduct pre-program staff survey*	45 (33.3%)	29 (25.7%)
	3.1 Create and maintain a support network	13 (9.6%)	12 (10.6%)
	3.2 Hold a well-being committee workshop	9 (6.7%)	8 (7.1%)
	**3.3 Hold a staff consultation workshop**	12 (8.9%)	11 (9.7%)
Do (Step 4)	4.1 Develop an Action Plan and launch	8 (5.9%)	7 (6.2%)
	**4.2 Promote with posters**	11 (8.1%)	10 (8.8%)
	**4.3 Promote with email reminders**	9 (6.7%)	8 (7.1%)
	4.4 Encourage change champions	5 (3.7%)	4 (3.5%)
Review (Step 5)	**5.1 Conduct follow-up workplace audit**	11 (8.1%)	8 (7.1%)
	5.2 Conduct follow-up staff survey*	19 (14.1%)	8 (7.1%)
	**Obtained evaluation data (pre & post surveys)**	9 (6.7%)	7 (6.2%)
	5.3 Reflect how the program went	5 (3.7%)	5 (4.4%)
Maximum phase reached	Pre-program	48 (35.6%)	46 (40.7%)
	Plan	60 (44.4%)	51 (45.1%)
	Do	4 (3.0%)	4 (3.5%)
	Review	23 (17.0%)	12 (10.6%)
**All Phases**	Business case**	16 (11.9%)	15 (13.3%)
Materials downloaded	Program overview**	87 (64.4%)	76 (67.3%)
	Background material**	12 (8.9%)	11 (9.7%)
	Staff survey example	26 (19.3%)	26 (23.0%)
	1.1 Business case template	64 (47.4%)	57 (50.4%)
	1.2 Sample policy	58 (43.0%)	52 (46.0%)
	2.2 Sample staff emails	52 (38.5%)	45 (39.8%)
	3.1 Committee invitation template	5 (9.4%)	5 (10.6%)
	3.2 Planning tool for workshop committee	23 (17.0%)	21 (18.6%)
	3.3 Email templates	22 (16.3%)	19 (16.8%)
	4.1 Action plan	27 (20.0%)	25 (22.1%)
	4.2a Tips for staff	47 (34.8%)	43 (38.1%)
	4.2b Posters	45 (33.3%)	43 (38.1%)
	4.3a Email guide	25 (18.5%)	22 (19.5%)
	4.3b Email templates	25 (18.5%)	22 (19.5%)
	4.4 Guide for change champions	15 (11.1%)	13 (11.5%)
	5.2 Follow-up staff survey email template and poster	10 (7.4%)	8 (7.1%)
	5.3 Replace workshop invite, PowerPoint and certificate	7 (13.2%)	5 (10.6%)

**Bolded =** core element of program (was highlighted in the toolkit as a core element)

* either data obtained, or step recorded as performed but no staff responses obtained

** These materials are accessible to all without a login and therefore incompletely tracked, unlike the other materials, which are only accessible with a login

### Engagement

**Fig. 2:**
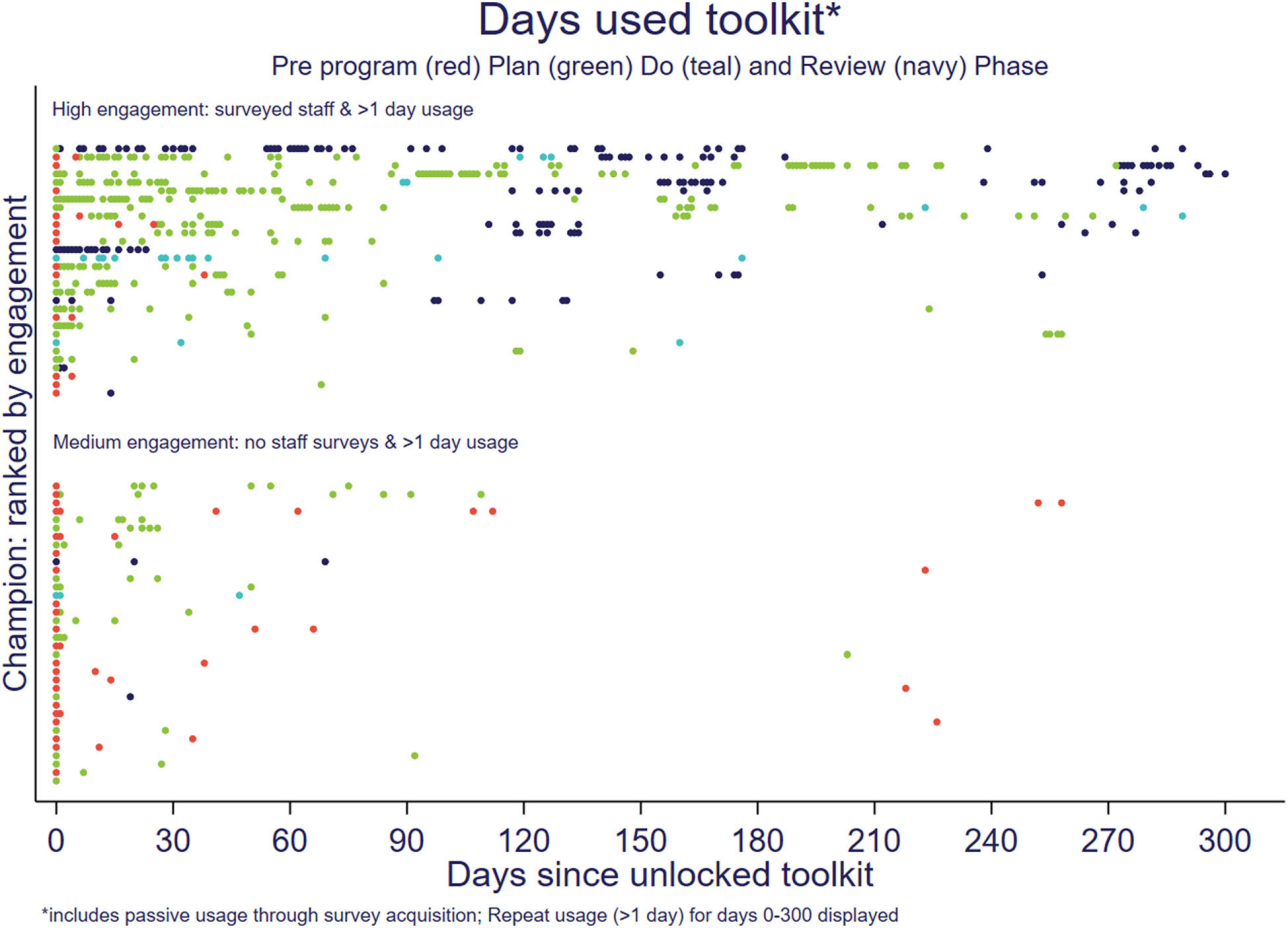
Engagement over the first 300 days since unlocking the toolkit in highly and moderately engaged champions

Champion toolkit usage spanned a timeframe as short as a single day to use that was still ongoing at the data census (> 300 days since unlocking toolkit). In total, 69 champions (51.1%) used the toolkit for a single day only and were considered low engagement, and 59 (52.1%) of combined teams had only low engagement champions. Figure 2 plots each of the moderately and highly engaged champions’ toolkit usage over time for the first 300 days since unlocking the toolkit. The amount and timing of when the champions engaged with the toolkit was highly varied, as were the extent and rate at which champions progressed through the program phases. The number of days of toolkit usage varied from 1 to 52 days with a median of 1 day, with this data largely indicative of how far into the program champions had progressed. Median (minimum, maximum) days of usage were: 1 (1,7) among champions who stopped before the ‘Plan’ phase; 1 (1,17) among those stopping during planning; 15 (3, 25) among those who stopped in the ‘Do’ phase; and, 12 (1, 52) in those who had reached the ‘Review’ phase.

Additional File 2 shows the engagement of staff with the staff surveys. In total, eight organisations who had only low-engagement champions sent out pre-program surveys, and always received back 0 or 1 staff responses — potentially filled in by the user themselves. Only the organisations with highly engaged champions sent out post-program surveys. These organisations received back between 0 and 122 responses to the staff pre-program survey (median = 30), representing a median estimated response rate relative to combined team sizes of 43%. There were 0 to 58 responses (median = 21.5) to the post-program survey, achieving a median estimated response rate of 34%. Team sizes were a problematic denominator for estimating response rates, sometimes being less than the number of responses (response rate > 100%) and in one instance being zero (response rate could not be calculated for the 11 respondents).

### Effectiveness

Table 5 shows changes in staff-reported sitting, standing and moving at work following the BeUpstanding program according to the evaluation data obtained from seven organisations (18 champions). Workplace sitting reduced on average by 9%, amounting to 43 min less sitting across an 8-h workday (− 9.0, 95% CI: − 12.0, − 5.9%). Correspondingly, there were statistically significant increases in standing time, and to a lesser extent in moving time. Results were largely unchanged in adjusted models and in the complete-case analysis. Variability between teams in their sitting changes was modest (ICC = 0.013, *p* = 0.3859) with average changes ranging between approximately 1% below and 1% above the overall mean change of 9% (Additional File 3).

**Table 5:** Program effectiveness on workplace sitting and physical activity in evaluation group ^a^

Model	Mean difference (95% CI)	p
**Evaluable cases**		
***Unadjusted (n = 337 pre, 167 post)***		
% Sitting	−9.0 (−12.0, −5.9)	< 0.001
% Standing	6.8 (4.2, 9.3)	< 0.001
% Moving	2.1 (0.6, 3.7)	0.007
***Adjusted for age & sex (n = 334 pre, 167 post)***		
% Sitting	−9.1 (−12.2, −6.1)	< 0.001
% Standing	6.8 (4.3, 9.3)	< 0.001
% Moving	2.2 (0.7, 3.8)	0.005
***Adjusted (n = 334 pre, 167 post)*** ^b^		
% Sitting	−9.2 (−12.3, −6.2)	< 0.001
% Standing	6.7 (4.2, 9.3)	< 0.001
% Moving	2.3 (0.8, 3.9)	0.003
**Complete cases**		
***Unadjusted (n = 73 pre, 73 post)***		
% Sitting	−9.2 (−13.1, −5.3)	< 0.001
% Standing	6.7 (3.5, 10.0)	< 0.001
% Moving	2.4 (0.4, 4.5)	0.017

Table reports mean difference (post vs pre-program) from linear mixed models, with random intercepts for cluster (organisational teams) and staff, unstructured covariance

^a^ Includes all organisational teams (teams from same organisation combined) with activity data collected in ≥1 pre- and ≥ 1 post- program staff survey: 7 organisational teams with 18 champions

^b^ Adjusted for age (years), sex (female yes/no), education (university yes/no) and employment (full time / part time / casual)

### Quality of data collection

Additional File 4 shows the data quality ratings for Phase 3 and the associated improvements made for Phase 4. Though collection procedures were suitable for many evaluation measures (especially for effectiveness), it was identified that several indicators were not fit-for-purpose. Correspondingly, improvements were made to achieve more robust data on key indicators of adoption and reach (especially verifying the user’s intention to champion the program and how many team members will be exposed to the program). Measures were also added to collect long-term outcomes, adverse events, costs, and non-participation / withdrawal data. The modifications made to address these deficits were chiefly adding additional surveys (maintenance; program completion) and items to surveys (adverse events; further team characteristics), as well as providing the user the ability to update their team numbers and state their intentions regarding their use of the toolkit and running the BeUpstanding program. Mostly these modifications were to the toolkit’s data collection procedures, rather than external researcher-driven mechanisms that would cease after the implementation trial.

## Discussion

This study reported on the findings from the early adopters phase of the BeUpstanding program, with the RE-AIM framework used to understand the extent to which the program was working as intended, and whether the evaluation was fit-for-purpose for a national implementation trial. Overall, there was good adoption of the program, with champions signing up from work teams across Australia and across multiple industries. The program was also found to be effective in the minority of teams completing the evaluation, with significant reductions in workplace sitting time achieved. However, implementation and engagement with the program varied widely amongst users with very few reaching the final review stage of the program. It was also identified that modifications were required to the evaluation protocol to ensure it was fit-for-purpose for the national implementation trial, with indicators of reach, implementation and maintenance particularly poorly collected. These learnings were used to optimise the program prior to Phase 4, the national implementation trial.

Findings showed that the targeted (but limited) partner promotional activities, informational boarding pages, and sign-up form worked sufficiently well to recruit diverse work teams (according to geography and industry). Furthermore, based on the data collected, the majority (81%) of those unlocking the toolkit would meet the basic screening criteria to participate in the implementation trial, suggesting the recruitment channels were appropriate. Importantly, the program was adopted by work teams across all five target sectors identified by the national regulators and state-based partners as being ‘at need’. Of note is that call centres, blue collar and small business teams were generally comprised of comparatively small team sizes, indicating that over sampling of these groups, as well as targeted recruitment, may be necessary to ensure adequate representation of these priority groups. It was identified that not all users were champions, and some users were working as part of a larger group rather than individually with a team. Team size, ranging from 0 to 8600, appears to be measured inaccurately, leading to difficulty in gauging reach and response rates accurately without direct contact of researchers with staff, as well as casting some doubt that the ‘team’ about whom the champion reported information are in fact the people who will go on to receive the BeUpstanding intervention. To address these limitations, functionality was added prior to Phase 4 to identify different roles of toolkit usage (e.g., workplace champion, senior decision maker) and to link related users, while team size was added to the report display in the online survey portals and users were given the ability to modify this information. Additionally, research staff now proactively monitor multiple registrations from the same organisation and, for those participating in the implementation trial, check and confirm the team size.

There was wide variation in program implementation and the extent to which users engaged with the toolkit and associated resources, with only a small number of toolkit users (< 10%) implementing and engaging with the program as intended. Specifically, despite the toolkit following a step-by-step process, most users did not complete the steps in order over the recommended 2–3 month program period and approximately half of users only logged on for single day. Similarly, despite being ‘flagged’ as important, core components were not completed systematically. Critically, data for some of these core elements, including the strategies that the work team participatively chose to sit less and move more, were not being captured. Intervention fidelity is a common issue facing evidence-informed programs in practice [28] and can be related to program design issues [29]. These findings highlighted a need to potentially be more prescriptive and provide incentives for correct implementation, as well as ensure that there were appropriate data capture mechanisms. Correspondingly, modifications for Phase 4 included: shifting the flexible program timeframe into a structured 8-week program; developing and embedding bespoke reporting into the toolkit to encourage and reward step completion; and, increasing data capture through additional surveys and survey items. Support for implementation was also added as part of the implementation trial to ensure completion of these core-components. Given that readiness for innovation has shown to be a key element of implementation [30], this variation in engagement and implementation may also be reflective of the stage of readiness for a program like BeUpstanding. Other elements likely to impact on program implementation include the level of support from leaders within the organisation for health promoting activities [31], and the characteristics of the champions delivering the program [32]. To understand this further, additional survey items were added prior to Phase 4 to capture organisational readiness [33], leadership support and champion characteristics.

Engagement with the evaluation components was mixed, with relatively good completion of the pre-program workplace audit by the user (> 50%), but poor distribution of the staff surveys, with less than 7% having both pre- and post-program staff data. The findings confirmed the effectiveness of the program (for those who do the program and evaluate it), with an average reduction in self-reported workplace sitting time of 43 min per workday. This change is line with findings reported from both the pilot study of BeUpstanding [22] and a meta-analysis of workplace interventions to reduce sedentary time [5]. With limited participation in the evaluation, it is not expected that these effectiveness findings would generalise to teams with less engaged champions who implement the program poorly. To improve adherence with the evaluation elements, customised, data-driven reports were developed and embedded into the toolkit, including a program completion report. This report, which only becomes available once all evaluation components are sufficiently completed, integrates information from multiple data sources to provide a bespoke report for the participating team on the impact and costs of the program. Researcher support for data collection, including support for how to promote the staff surveys, was also added as part of the implementation trial.

### Strengths and limitations

The deliberate inclusion of this early adopters phase, with associated soft launch, into the planned translation of the BeUpstanding program is a key strength as piloting the protocol in a “real-world” context provides an opportunity to refine research methods prior to wide-spread promotion [34]. This step is recommended for ensuring the feasibility of broader implementation, as well as reducing costs and minimising potential harms, but it is often missed [34, 35]. Another key strength of this study was the use of RE-AIM [18]: a framework widely used in health research and shown to be an efficient tool for evaluating community-based projects [36]. Innovatively, RE-AIM was used to not only evaluate the program but to also evaluate the extent to which the toolkit and data collection procedures were “fit-for-purpose” for the planned national implementation trial. Other strengths include the detailed collection of engagement data to understand how users were interacting with the toolkit, and the participation from a wide range of target end-users from across Australia, albeit not a representative or balanced sample of users. Limitations include the self-report of many of the key indicators, including the behavioural outcomes. Additionally, as identified, some of the core steps of the program were not captured so it was unclear the extent to which users were engaging in those elements. Furthermore, although much of the engagement data was automatically captured through the toolkit analytics, it is unknown the extent to which users were engaging with any of the materials offline.

In conclusion, findings from this early adopters phase provided important practice-based evidence on the extent to which the BeUpstanding program was working as intended and whether the data collection procedures were fit-for-purpose for a national implementation trial. They highlight the importance of this phase in the translation process and provide an example of how the learnings can be used for iterative improvement to maximise the success of research to practice.

## Supplementary Information

**Additional file 1.**

**Additional file 2.**

**Additional file 3.**

**Additional file 4.**

## Data Availability

De-identified datasets generated and analysed during the current study are available in the UQ eSpace repository (10.48610/8f73a27).
