# Supplementary material for: A RE-AIM evaluation in early adopters to iteratively improve the online BeUpstanding™ program supporting workers to sit less and move more
Source: BMC Public Health. 2021 Oct 22;21:1916. doi: 10.1186/s12889-021-11993-1 (PMC8532381; doi:10.1186/s12889-021-11993-1)
Supplement: Supplementary file 1 — Additional file 1. [file 12889_2021_11993_MOESM1_ESM.docx]

**Additional File 1:** Number of steps performed by each individual champion and by at least one champion for each combined team

| Number of steps performed (after unlocking toolkit) | Champions  n (%) of 135 | Teams  n (%) of 113^a^ |
| --- | --- | --- |
| 0 | 48 (35.6%) | 46 (40.7%) |
| 1 | 27 (20.0%) | 20 (17.7%) |
| 2 | 17 (12.6%) | 13 (11.5%) |
| 3 | 13 (9.6%) | 8 (7.1%) |
| 4 | 4 (3.0%) | 11 (9.7%) |
| 5 | 3 (2.2%) | 3 (7.1%) |
| 6 | 1 (0.7%) | 3 (7.1%) |
| 7 | 0 (0.0%) | 0 (0.0%) |
| 8 | 1 (0.7%) | 1 (0.9%) |
| 9 | 2 (1.5%) | 2 (1.8%) |
| 10 | 3 (2.2%) | 3 (7.1%) |
| 11 | 0 (0.0%) | 0 (0.0%) |
| 12 | 1 (0.7%) | 0 (0.0%) |
| 13 | 1 (0.7%) | 1 (0.9%) |
| 14 | 2 (1.5%) | 2 (1.8%) |

^a^ Performed for combined team by one or more champions in organisation
