# Supplementary material for: A RE-AIM evaluation in early adopters to iteratively improve the online BeUpstanding™ program supporting workers to sit less and move more
Source: BMC Public Health. 2021 Oct 22;21:1916. doi: 10.1186/s12889-021-11993-1 (PMC8532381; doi:10.1186/s12889-021-11993-1)
Supplement: Supplementary file 2 — Additional file 2. [file 12889_2021_11993_MOESM2_ESM.docx]

**Additional File 2:** Staff engagement with the surveys in organisations who sent out surveys, by champion engagement with the toolkit

| Survey | Statistic | High engagement teams^a^ | Low engagement teams |
| --- | --- | --- | --- |
| Pre survey | N organisations | n=21 | n=8 |
|  | Combined team size | 30 (0, 305) | 42.5 (20, 300) |
|  | N responses | 6 (0, 122) | 0 (0, 1) |
|  | Estimated Response Rate ^b^ | 43% (0%, 120%) ^c^ | 0% (2%, 3%) |
| Post survey | N organisations | n=8 | n=0 |
|  | Combined team size | 41 (5, 305) | - |
|  | N responses | 21.5 (0, 58) | - |
|  | Estimated Response Rate ^b^ | 34% (0%, 64%) | - |

Table displays median (min, max).

^a^ ≥1 high engagement champion within organisation

^b^ Total number of responses for the organisation divided by total combined team size for the organisation, excluding organisations with combined team size = 0
