# Supplementary figures and images for: A RE-AIM evaluation in early adopters to iteratively improve the online BeUpstanding™ program supporting workers to sit less and move more
Source: BMC Public Health. 2021 Oct 22;21:1916. doi: 10.1186/s12889-021-11993-1 (PMC8532381; doi:10.1186/s12889-021-11993-1)

**Additional File 3**: Variation by teams in % sitting changes (Best Unbiased Linear Predictions)


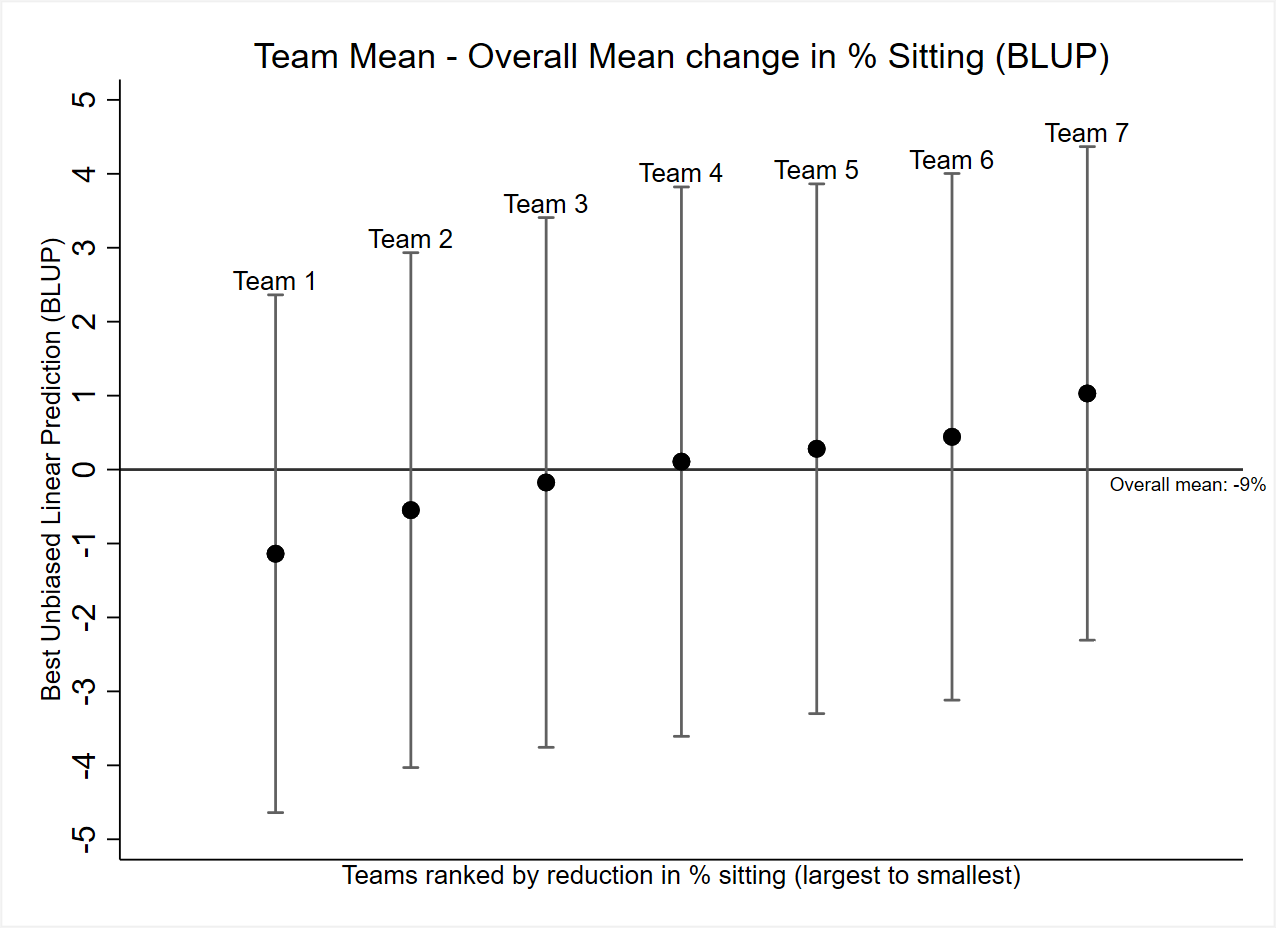

Supplement: Supplementary file 3 — Additional file 3. [file 12889_2021_11993_MOESM3_ESM.docx]
