# Supplementary material for: A RE-AIM evaluation in early adopters to iteratively improve the online BeUpstanding™ program supporting workers to sit less and move more
Source: BMC Public Health. 2021 Oct 22;21:1916. doi: 10.1186/s12889-021-11993-1 (PMC8532381; doi:10.1186/s12889-021-11993-1)
Supplement: Supplementary file 4 — Additional file 4. [file 12889_2021_11993_MOESM4_ESM.docx]

**Additional File 4**: Quality of data collection in Phase 3 according to the indicators of RE-AIM, and modifications made to ensure the evaluation was fit-for-purpose for the national implementation trial (Phase 4).

| **Data needed to enable evaluation according to the indicators of RE-AIM** | **Phase 3** | **Modifications for Phase 4** | **Phase 4** |
| --- | --- | --- | --- |
| **Reach of staff in teams** |  |  |  |
| Staff in work team (n as reported by champion) |  | Champion able to modify team size in their profile | *** |
| % of staff in work team that participate in choosing BeUpstanding strategies |  | Captured in new hard-coded survey in toolkit |  |
| n (%) participation in staff surveys (n unique responses / team size) |  | Increased confidence with denominator; added participation rate into real-time report |  |
| Characteristics of staff taking part in the evaluation |  | --- |  |
| Number and characteristics of non-participating staff |  | Basic team details reported by champion as part of workplace audit |  |
| Number of staff that withdraw and reasons for withdrawal |  | Option for reason for non-participation added to staff survey; engagement by staff reported by champion in new end-of-program survey |  |
| **Effectiveness** |  |  |  |
| Workplace sitting and activity |  | --- |  |
| Activity preference alignment |  | --- |  |
| Organisational social norms |  | --- |  |
| Enablers to sitting less and moving more |  | --- |  |
| Perceived barriers to sitting less and moving more |  | --- |  |
| Work performance and engagement |  | --- |  |
| General health |  | --- |  |
| Adverse / unintended consequences (end program only) for champions |  | Captured in new end-of-program survey |  |
| Adverse / unintended consequences (end program only) for staff |  | Question added into post-program staff survey |  |
| Costs to deliver the BeUpstanding program |  | Captured in new end-of-program survey |  |
| Program satisfaction and perceived impact (end program only) for champions |  | Captured in new end-of-program survey |  |
| Program satisfaction and perceived impact (end program only) for staff |  | --- |  |
| **Adoption by Teams** |  |  |  |
| Champions registering for BeUpstanding (n) |  | Question added to champion profile survey clarifying the role of person completing survey |  |
| Champions unlocking the toolkit (n) |  | --- |  |
| Characteristics of champions and their organisations and their work teams (including size of organisation and number of staff) |  | --- | *** |
| Reasons for taking up the program |  | --- |  |
| Champions eligible and enrolling in implementation trial (n, % of eligible) |  | --- |  |
| Champion withdrawals from implementation trial (n) and reasons for withdrawal |  | Captured by research team only (not in toolkit) | *** |
| **Implementation** |  |  |  |
| Completion rates |  | Confirmed through new end-of-program survey | *** |
| Engagement with the program |  |  |  |
| Strategies chosen by work team |  | Captured in new hard-coded survey in toolkit | *** |
| Sit less, move more strategies (staff) |  | --- |  |
| Barriers and enablers to implementation |  | Captured in new end-of-program survey |  |
| **Maintenance** |  |  |  |
| Self-reported workplace sitting time collected 9-months after end-of program |  | Added survey for staff into toolkit (unlocked after completion of post-program survey) |  |
| Use of activity policies and practices |  | Added follow-up workplace audit plus automated report |  |

Key: red: not captured at all; orange: captured but issues with accuracy / validity; green: adequate / fit for purpose for implementation trial; --- no change made

*** data confirmed by research team as part of the implementation trial
